# Cone Beam Computed Tomographic Analyses of the Position and Course of the Mandibular Canal: Relevance to the Sagittal Split Ramus Osteotomy

**DOI:** 10.1155/2014/945671

**Published:** 2014-02-27

**Authors:** Ahmet Ercan Sekerci, Halil Sahman

**Affiliations:** ^1^Department of Oral and Maxillofacial Radiology, Faculty of Dentistry, Erciyes University, 38039 Kayseri, Turkey; ^2^Department of Oral and Maxillofacial Radiology, Faculty of Dentistry, Abant Izzet Baysal University, 14280 Bolu, Turkey

## Abstract

*Purpose*. The aim of this study was to document the position and course of the mandibular canal through the region of the mandibular angle and body in dental patients, using cone beam computed tomographic imaging. *Methods*. The position and course of the mandibular canal from the region of the third molar to the first molar were measured at five specific locations in the same plane: at three different positions just between the first and second molars; between the second and third molars; and just distal to the third molar. *Results*. The study sample was composed of 500 hemimandibles from 250 dental patients with a mean age of 26.32. Significant differences were found between genders, distances, and positions. B decreased significantly from the anterior positions to the posterior positions in both females and males. The mean values of S and CB increased significantly from the posterior positions to the anterior positions in both females and males. *Conclusion*. Because the sagittal split ramus osteotomy is a technically difficult procedure, we hope that the findings of the present study will help the surgeon in choosing the safest surgical technique for the treatment of mandibular deformities.

## 1. Introduction

The most widely used orthognathic surgical method of the mandible for the correction of dentofacial deformities is the sagittal split ramus osteotomy (SSRO) [1]. Trauner and Obwegeser [2] popularized this technique in 1957 for the correction of prognathism and retrognathism. Since then, SSRO has become the standard procedure in the treatment of mandibular deformity [3–7]. In this technique, the mandibular ramus is split on both sides in the sagittal plane, and the distal fragment is moved forward or backward to correct the bite [8].

Understanding the detailed anatomy of the inferior alveolar canal (IAC) is essential for dental practitioners to avoid potential injury to the nerve during surgical procedures [9]. Due to the position and course of the mandibular canal, the inferior alveolar nerve (IAN) is at great risk of injury during SSRO [8]. Postoperative neurosensory disturbances can result from traction on the IAN during surgery, trauma to the nerve during splitting, and incorrect placement of bone screws during the rigid fixation stage [10]. Cutting instruments may also cause direct injury to the nerve at the vertical osteotomy stage, due to the close relationship of the IAC to the buccal cortex and/or to the inferior border of the mandible.

The present study was designed to determine the position of the mandibular canal with respect to the external surfaces of the mandible (buccal, lingual, superior, and inferior). This study also aimed to describe the variability of the mandibular canal within the angle and body in order to determine the safest site and distance for the SSRO through the buccal surface, lingual cortical plate, and inferior border.

## 2. Materials and Methods

A retrospective study was performed using the cone beam computed tomography (CBCT) mandibular image records of 250 dental patients at the Department of Oral and Maxillofacial Radiology, Faculty of Dentistry, Erciyes University, Kayseri, Turkey. The patients had required the CBCT images due to various dental problems (e.g., implant placement, surgery planning, orthodontic treatment, and pathosis). The CBCT examinations, which automatically set the appropriate exposure parameters for each patient, were conducted using a NewTom 5G CBCT scanner (QR, Verona, Italy).

Inclusion criteria in this study sample were as follows:

- absence of any developmental disturbance, pathology, or previous treatment that could influence the IAC, canal, or tooth position, including impactions,
- complete set of mandibular molar teeth,
- radiographically completely bilaterally corticated IAC canal,
- absence of radiological evidence of skeletal/dental malocclusion that could have altered the positions of molar teeth or IAC,
- age of patient ≥ 18 years.

The CBCT scans were analyzed by two independent, experienced oral radiologists (AES and HS). The CBCT images were analyzed with built-in software (NNT) in a Dell Precision T5400 workstation (Dell, Round Rock, TX) with a 32-inch Dell LCD screen with a resolution of 1280 × 1024 pixels in a darkroom. The contrast and brightness of the images were adjusted using the image processing tool in the software to ensure optimal visualization. Tomography positions of 0.25 mm in the multiplanar images were created. Using the axial, coronal, and sagittal sections, the exact locations of the IAC and tooth were identified for the study. These images were transmitted to a personal computer in the digital imaging and communications in medicine (DICOM) format and reconstructed into multiplanar reconstruction images using a DICOM viewer (ExaVision SX version 1.13; Ziosoft Inc., Tokyo, Japan). Values were measured individually by the authors. The mean of the values was considered the measurement for the particular patient. Kappa values between observers ranged between 0.93 and 0.82 for all values.

**Figure 1:**
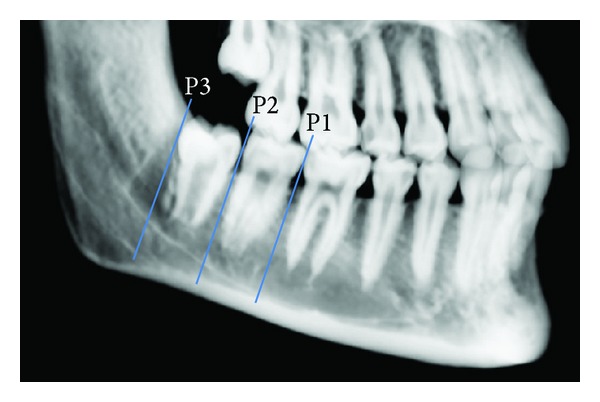
Diagram showing the sites of the three positions (P1, P2, and P3) through the mandible. The coronal computed tomography scans were made perpendicular to the mandibular occlusal plane.

**Figure 2:**
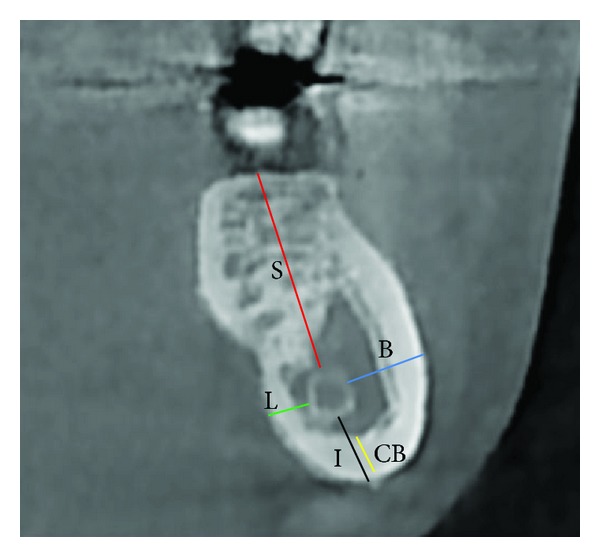
Diagram of various linear measurement parameters showing the distance from the outer surface of the mandibular canal to the buccal (B), lingual (L), superior (S), and inferior (I) surfaces of the mandible and the thickness of inferior cortical bone (CB).

For each patient, one scan was taken and three points were measured at the following levels: P1, just between the first and second molars; P2, between the second and third molars; and P3, just distal to the third molar (Figure 1). The following variables were measured: distance between the external surface of the buccal cortical plate and the outer surface of the mandibular canal (B); distance between the external surface of the lingual cortical plate and the outer surface of the mandibular canal (L); distance between the external surface of the inferior border of the mandible and the outer surface of the mandibular canal (I); distance from the superior aspect of the canal to the alveolar crest (S); and thickness of the inferior cortical bone (CB) (Figure 2).

All the data were entered and analyzed using SPSS, version 16 (SPSS Inc., Chicago, IL). Descriptive statistics of the variables and measurements are presented. Probabilities ≤ 0.05 were accepted as significant.

## 3. Results

The study subjects consisted of 121 (48.4%) males and 129 (51.6%) females. The mean age of the patients was 26.32 (SD: 6.34), with ages ranging from 18 to 40 years.  Table 1 depicts the mean value of the linear measurements of parameters in the study population as well as the differences in terms of gender among the various parameters.

Distance B decreased significantly from the anterior positions to the posterior positions (1, 2, and 3, resp.) in both females (right side: 6.4 ± 1.53, 5.70 ± 1.57, and 4.0 ± 1.61; left side: 6.4 ± 1.67, 5.8 ± 1.85, and 4.1 ± 1.79) and males (right side: 6.3 ± 1.85, 5.6 ± 1.57, and 3.6 ± 1.73; left side: 6.6 ± 1.38, 5.9 ± 1.60, and 3.9 ± 1.48).

Distance L was significantly greater at position 3 than at positions 1 and 2 in both females (2.63 ± 1.41, 2.38 ± 0.92, and 2.32 ± 0.88) and males (2.70 ± 1.32, 2.21 ± 0.99, and 1.92 ± 0.92), from posterior to anterior (*P* < 0.01).

Distance S was also significantly different between genders in both positions, except in the third position on the left side. CB measurements increased significantly from the posterior positions to the anterior positions in both females (2.88 ± 0.83, 3.18 ± 0.54, and 3.34 ± 0.57) and males (2.89 ± 0.76, 3.25 ± 0.48, and 3.50 ± 0.61) (*P* < 0.01).

The age group breakdown and number of subjects are shown in Tables 2(a)–2(c). In patients from 18 to 25 years of age, distance I was significantly different between genders in both positions (*P* = 0.000).

## 4. Discussion

BSSRO is a well-established procedure, and many authors have attempted various modifications to the surgical technique [11]. Patients undergoing SSRO are mostly young individuals, and as they have a mandibular deficiency, prognathism, and asymmetry, it is likely that the rami anatomy of these patients is different from that of typical cadaveric specimens [8].

Several modifications of the technique have been introduced, with the aim of improving surgical convenience, minimizing morbidity, and maximizing procedural stability [1]. These modifications include the technique described by Dal Pont [12]; it is generally recognized that the buccal osteotomy cut of the Obwegeser-Dal Pont method is positioned more anteriorly than that of the Obwegeser method [13], thereby increasing the amount of cancellous bone contact. In the Trauner-Obwegeser technique, the lateral osteotomy cut is made horizontally from the distal region of the second molar to the posterior border, well above the mandibular angle [2]. This osteotomy technique was first performed in 1955 [14]. In the Obwegeser technique, which was introduced in 1957, the lateral osteotomy cut is made from the distal region of the second molar to the midpoint of the mandibular angle. In the Obwegeser-Dal Pont technique, the lateral osteotomy cut is made vertically from distal region of the second molar to the lower border of the ascending ramus. This osteotomy technique was first performed in 1958 [14]. In orthognathic surgery of the mandibular ramus, intraoperative complications such as lesions of the inferior alveolar nerve, fractures of the osteotomized segments, incomplete sectioning, malpositioning of segments, and hemorrhage may occur [15].

Knowledge of the anatomy of the mandibular canal and its related structures can provide information regarding the degree and extent of damage to the IAN resulting from both direct and indirect injuries [16]. Previous studies have yielded a 12.5%–100% postoperative incidence rate of sensory deficits of the IAN immediately after surgery. Long-term follow-up has shown a 0%–85% incidence rate of sensory alteration one or two years after surgery [17]. To reduce injuries to the IAN during SSRO, knowledge of the anatomic location and course of the mandibular canal is imperative [8]. Although anatomic studies of the mandibular canal have been performed in terms of the position and course of the mandibular canal for the performance of SSRO [8, 10, 18], only Tsuji et al. [8] described the anatomic variability of the mandibular canal within the rami to assist in determining the safest site for a vertical corticotomy through the buccal plate when splitting the mandible.

Few anatomic studies have observed the bone thickness of the mandible at possible SSRO osteotomy sites. If the patient has a thin ramus, the sagittal splitting technique involves the risk of a bad split or neurologic injury. It has also been shown, however, that vascular and nerve bundles may be extremely close to the buccal cortex of the mandible in a broad and thick ramus [19]. This was observed in only 6% (10/164) of the mandibles in a study reported by Tamas [20].

In a study by Ylikontiola et al. [21], the mandibular canal was in direct contact with the buccal cortex of the mandible in 7% (3/40) of the mandibular sides. In their study, the distance from the mandibular canal to the lateral border of the mandible was observed to be 3.5 mm (ranging from 1.8 to 6.5 mm) between the first and second molars and 2.5 mm (ranging from 0.4 to 5.9 mm) distal to the third molar. Similar to their results, Rajchel et al. found that the greatest distance between the cortical plate and the mandibular canal was at the level of the first and second molars, while the smallest was at the third molar [22]. Tsuji et al. [8] found that 16 of 70 rami (22.9%) had this contact or fusion type of mandibular canal, and it was often observed from the mandibular foramen to the mandibular angle. Yamamoto et al. [10] found that the mandibular canal came into contact with the external cortical bone in 10 of 40 rami (25%). Their study showed that neurosensory disturbance was significantly more likely to be present one year after surgery when the width of the marrow space between the mandibular canal and the external cortical bone was 0.8 mm or less. However, that study did not clarify the entire course of the mandibular canal from the mandibular foramen to the mandibular body. Ueki et al. [19] suggested that the horizontal distance between the mandibular canal and the lateral cortex in the mandibular foramen level was made by SSRO with a bent plate fixation.

With all the previous modifications, a constant finding has been formed that a split usually does not occur at the inferior border of the mandible, but rather, on the lingual aspect of the mandible, somewhere between the inferior border and the superior aspect of the IAC. When the fractures occur on the medial aspect of the ramus above the level of the cortical bone of the inferior and/or around the level of the neurovascular bundle, it is virtually impossible to place a bone screw below the level of the IAN. If there is inadequate vertical cutting of the inferior border, there is still a risk that a buccal cortical fracture or a standard medial fracture above the inferior border might occur [23]. A study by Chen et al., performed using horizontal CT images, showed that the distance between the mandibular canal and the split surface correlated with trigeminal somatosensory-evoked potential latency recovery [9].

Reducing the risk of damage to anatomical structures such as nerves, vessels, and neighboring structures is one of the desired outcomes of preoperative computer-aided planning [24, 25]. CBCT has been reported as a well-suited advanced imaging modality for the maxillofacial area. It provides clear and accurate images of structures and, therefore, is extremely useful for assessing bone components. As the resultant images displayed are often corrected for magnification, accurate measurements can be derived from the reformatted three-dimensional data [26]. Computer-assisted navigation, which allows real-time imaging of the surgical drill as an overlay graphic on the CT and live intraoperative video images, has been reported as suitable for routine clinical applications [27]. When Park et al. evaluated the difference of the midfacial soft-tissue changes between groups, CBCT superimposition was utilized, and the soft-tissue postoperative changes were measured [28]. They stated that to resolve the limitations of 2D image superimposition, CBCT may be a good tool for the assessment of treatment outcomes, because the clinician can simultaneously view the soft and hard tissues using new superimposition techniques. In another study, on Korean subjects who had undergone mandibular setback surgery by SSRO, skeletal stability was evaluated by Ghang et al. [29] with a lateral cephalogram and three-dimensional CBCT. Kim et al. [30] carried out a study to compare the short- and long-term changes in condylar position related to the glenoid fossa, skeletal, and occlusal stability after orthognathic surgery. In all these studies, the patients were assessed by CBCT images for condylar rotational changes and anteroposterior position in the presurgery, postsurgery, and postretention periods. In the present study, we performed a retrospective study using CBCT images. Three different positions and five distances were selected for the measurement of bone thickness with respect to the IAC. In accordance with the previous studies, the mean distance from the buccal cortex to the IAC increased significantly from the posterior planes to the anterior planes. Although the mean distance from IAC to the buccal cortex at the second molar region was found to be smaller in females [31], this distance was not influenced by the gender of patients in our study. In the present study, the greatest I distance was observed to be distal to the third molar, and it did not differ among more anterior positions in males. However, this distance gradually decreased from the posterior positions to the anterior positions in females. In contrast, CB thickness decreased from the posterior positions to the anterior positions in both genders. Contrary to our previous inference, making a vertical cut distal to the third molar may decrease the incidence of direct injury to the IAN. The vertical osteotomy is the safest when it is performed between the second and third molars, considering the bone thickness both buccally and inferiorly to the IAC.

In conclusion, the results of the present study have demonstrated the anatomic position of the IAC through the region of the mandibular angle and body in dental patients, using CBCT imaging. This study suggests that the second position seems to be the safest site considering the adequate bone distances. Our results also suggest that to detect the position and course of the IAC and bone thickness at possible osteotomy sites, a CBCT survey must be carried out of patients who are candidates for SSRO.

## Figures and Tables

**Table 1:** Measurements in three different positions of the mandible with their comparison between gender-side in millimeters.

		Side	Gender	Minimum	Maximum	Mean	SD	*P* value	Total	
									Mean	SD
P1	B	Right	1	3.3	10.8	6.4	1.53	0.650	6.5	1.54
			0	2.2	9.9	6.3	1.85			
		Left	1	3.5	11.4	6.4	1.67	0.452	6.3	1.69
			0	3.6	10.6	6.6	1.38			
	L	Right	1	0.9	7.6	2.4	1.02	0.000*	2.2	1.00
			0	0.6	5.8	1.9	0.92			
		Left	1	1.0	4.0	2.2	0.70	0.004*	2.1	0.83
			0	0.8	5.0	1.9	0.94			
	I	Right	1	1.4	11.4	6.1	1.75	0.000*	6.7	1.75
			0	3.4	13.0	7.4	1.52			
		Left	1	3.1	12.1	6.5	1.58	0.000*	6.9	1.63
			0	3.3	13.0	7.3	1.57			
	S	Right	1	8.6	19.6	14.4	1.95	0.000*	15.2	2.47
			0	10.1	23.8	16.0	2.69			
		Left	1	11.0	19.7	15.1	2.08	0.000*	15.6	2.58
			0	11.1	24.0	16.2	2.92			
	CB	Right	1	1.7	4.8	3.4	0.56	0.011*	3.5	0.60
			0	2.4	5.9	3.6	0.63			
		Left	1	1.8	5.2	3.3	0.59	0.042*	3.4	0.60
			0	2.3	5.1	3.4	0.60			

P2	B	Right	1	2.7	9.1	5.7	1.57	0.428	5.7	1.57
			0	2.2	9.4	5.6	1.57			
		Left	1	1.5	12.8	5.8	1.85	0.511	5.8	1.73
			0	2.8	10.0	5.9	1.60			
	L	Right	1	0.8	5.1	2.5	0.96	0.172	2.4	1.01
			0	0.7	5.1	2.3	1.06			
		Left	1	0.8	4.7	2.3	0.88	0.166	2.2	0.90
			0	0.8	4.3	2.1	0.92			
	I	Right	1	2.9	14.0	7.0	1.80	0.000*	7.6	1.79
			0	4.8	12.4	8.2	1.57			
		Left	1	2.8	11.6	7.3	1.57	0.000*	7.7	1.76
			0	3.0	14.5	8.2	1.83			
	S	Right	1	6.1	19.7	10.6	2.21	0.000*	11.3	2.57
			0	6.5	20.2	12.2	2.66			
		Left	1	6.2	14.9	11.3	1.77	0.045*	11.6	2.05
			0	4.3	19.3	11.8	2.30			
	CB	Right	1	2.0	4.7	3.2	0.53	0.043*	3.2	0.51
			0	2.5	4.4	3.3	0.49			
		Left	1	1.9	4.9	3.2	0.57	0.735	3.2	0.52
			0	2.5	4.1	3.2	0.47			

P3	B	Right	1	0.8	9.4	4.0	1.61	0.041*	3.8	1.68
			0	1.1	9.3	3.6	1.73			
		Left	1	1.3	9.5	4.1	1.79	0.549	4.0	1.65
			0	1.3	8.5	3.9	1.48			
	L	Right	1	0.8	6.3	2.8	1.46	0.620	2.8	1.45
			0	0.6	7.3	2.9	1.45			
		Left	1	0.6	6.5	2.5	1.35	0.728	2.5	1.27
			0	0.7	6.8	2.6	1.19			
	I	Right	1	5.5	17.0	10.4	2.37	0.000*	11.1	2.48
			0	4.4	16.0	11.9	2.39			
		Left	1	5.4	16.8	10.7	2.12	0.000*	11.3	2.41
			0	3.0	18.2	12.0	2.51			
	S	Right	1	5.4	15.3	9.4	2.02	0.010*	9.7	2.11
			0	4.3	18.5	10.1	2.15			
		Left	1	3.8	13.3	9.7	2.05	0.095	9.9	1.95
			0	4.5	13.5	10.1	1.81			
	CB	Right	1	1.1	5.4	2.8	0.80	0.555	2.8	0.71
			0	1.8	5.5	2.9	0.60			
		Left	1	1.5	7.0	3.0	0.86	0.805	2.9	0.88
			0	2.1	8.0	2.9	0.90			

Groups: 1: women, 0: men; *: statistical significance; SD: standard deviation.

The distance between the external surface of the buccal cortical plate and the outer surface of the mandibular canal (B); between the external surface of the lingual cortical plate and the outer surface of the mandibular canal (L); between the external surface of the inferior border of the mandible and the outer surface of the mandibular canal (I); from superior aspect of the canal to the alveolar crest (S) and the thickness of inferior cortical bone (CB).

**Table:** (a)

Position		Side	Gender	Minimum	Maximum	Mean	SD	*P* value
P1	B	Right	0	4.0	9.5	6.92	1.55	0.257
			1	3.5	11.4	6.58	1.75	
		Left	0	3.3	9.9	6.78	1.60	0.489
			1	3.3	10.1	6.59	1.51	
	L	Right	0	0.9	5.0	1.94	1.01	0.056
			1	1.1	3.8	2.22	0.67	
		Left	0	1.0	5.8	2.21	1.00	0.469
			1	1.1	5.3	2.33	0.77	
	I	Right	0	3.3	13	7.32	1.96	0.000*
			1	3.1	9.4	6.26	1.34	
		Left	0	4.8	10.1	7.39	1.35	0.000*
			1	1.4	11.4	5.97	1.65	
	S	Right	0	2.4	4.7	3.45	0.54	0.063
			1	1.8	5.0	3.26	0.60	
		Left	0	2.4	4.8	3.49	0.58	0.370
			1	2.4	4.8	3.40	0.53	
	CB	Right	0	11.3	24.0	16.66	2.93	0.000*
			1	11.0	19.7	14.82	1.93	
		Left	0	13.0	23.8	16.76	2.52	0.000*
			1	8.6	19.6	14.39	1.86	

P2	B	Right	0	2.7	9.1	6.44	1.54	0.104
			1	1.5	12.8	5.93	1.88	
		Left	0	3.6	9.4	6.02	1.54	0.433
			1	2.8	10.0	5.80	1.63	
	L	Right	0	0.8	4.1	2.14	0.80	0.125
			1	0.8	4.7	2.38	0.92	
		Left	0	1.0	5.1	2.65	1.19	0.481
			1	1.3	5.1	2.52	0.88	
	I	Right	0	3.0	14.5	8.36	2.18	0.000*
			1	2.8	11.6	7.09	1.53	
		Left	0	5.5	11.6	8.43	1.49	0.000*
			1	2.9	14.0	6.93	1.93	
	S	Right	0	2.6	4.1	3.23	0.42	0.306
			1	2.0	4.9	3.13	0.57	
		Left	0	2.6	4.2	3.29	0.41	0.099
			1	2.1	4.7	3.14	0.54	
	CB	Right	0	8.3	19.3	12.06	2.36	0.040*
			1	6.2	14.9	11.31	1.77	
		Left	0	7.7	16.6	12.78	2.42	0.000*
			1	6.1	15.6	10.50	2.09	

P3	B	Right	0	1.6	7.0	4.35	1.28	0.484
			1	1.3	9.5	4.15	1.78	
		Left	0	1.3	9.3	3.80	1.81	0.326
			1	1.3	7.5	4.08	1.43	
	L	Right	0	0.7	5.6	2.61	1.22	0.523
			1	0.6	5.6	2.46	1.30	
		Left	0	0.9	7.3	3.19	1.58	0.042*
			1	0.8	6.0	2.66	1.34	
	I	Right	0	6.6	18.2	12.21	2.53	0.000*
			1	5.4	16.8	10.68	2.06	
		Left	0	4.4	16.0	12.11	2.38	0.000*
			1	5.5	17.0	10.42	2.33	
	S	Right	0	2.3	4.7	2.98	0.64	0.484
			1	1.5	5.9	2.89	0.81	
		Left	0	1.9	4.3	2.87	0.56	0.521
			1	1.1	5.4	2.78	0.83	
	CB	Right	0	6.9	13.5	10.41	1.77	0.065
			1	5.1	13.3	9.79	1.92	
		Left	0	7.3	18.5	10.65	1.91	0.000*
			1	5.4	15.3	9.39	1.86	

**Table:** (b)

Position		Side	Gender	Minimum	Maximum	Mean	SD	*P* value
P1	B	Right	0	3.6	8.0	6.07	1.08	0.976
			1	3.5	9.8	6.07	1.53	
		Left	0	2.2	9.9	5.93	2.18	0.795
			1	3.5	10.8	6.05	1.60	
	L	Right	0	0.8	4.8	1.86	0.77	0.070
			1	1.0	4.0	2.21	0.81	
		Left	0	0.6	3.3	1.60	0.70	0.000*
			1	1.1	7.6	2.60	1.27	
	I	Right	0	5.3	10.2	7.25	1.11	0.232
			1	3.3	12.1	6.84	1.77	
		Left	0	4.9	11.1	7.35	1.25	0.016*
			1	1.7	9.5	6.42	1.92	
	S	Right	0	2.4	4.7	3.43	0.60	0.683
			1	2.6	5.2	3.38	0.50	
		Left	0	2.8	5.3	3.76	0.63	0.009*
			1	1.7	4.5	3.36	0.61	
	CB	Right	0	11.1	24.0	16.06	3.22	0.219
			1	11.3	19.7	15.22	2.25	
		Left	0	13.1	23.8	15.90	2.91	0.027*
			1	11.2	19.1	14.49	2.13	

P2	B	Right	0	2.8	9.1	5.35	1.48	0.575
			1	3.0	8.7	5.16	1.45	
		Left	0	2.6	8.6	5.21	1.55	0.258
			1	3.0	8.9	5.63	1.59	
	L	Right	0	0.8	4.3	2.24	1.09	0.767
			1	1.0	3.6	2.17	0.77	
		Left	0	0.7	3.4	1.97	0.92	0.026*
			1	1.1	4.7	2.52	1.12	
	I	Right	0	5.2	11.4	7.99	1.39	0.442
			1	4.5	10.1	7.72	1.58	
		Left	0	6.3	12.4	8.16	1.38	0.010*
			1	3.9	10.2	7.20	1.66	
	S	Right	0	2.5	4.1	3.20	0.49	0.552
			1	2.2	4.5	3.27	0.49	
		Left	0	2.5	4.4	3.20	0.51	0.852
			1	2.1	4.0	3.22	0.42	
	CB	Right	0	4.3	17.4	11.85	2.48	0.255
			1	6.2	14.3	11.26	1.70	
		Left	0	7.5	20.2	12.25	2.74	0.016*
			1	6.1	19.7	10.57	2.93	

P3	B	Right	0	1.3	6.3	3.43	1.23	0.624
			1	1.3	7.8	3.59	1.62	
		Left	0	1.1	5.9	3.30	1.38	0.202
			1	1.6	9.4	3.78	1.82	
	L	Right	0	1.1	6.8	2.59	1.18	0.853
			1	0.6	6.5	2.65	1.31	
		Left	0	0.6	7.3	2.36	1.29	0.036*
			1	0.9	6.3	3.10	1.62	
	I	Right	0	3.0	15.5	11.82	2.50	0.176
			1	7.4	14.3	11.08	1.86	
		Left	0	9.1	15.6	12.15	1.96	0.001*
			1	5.7	15.3	10.40	2.45	
	S	Right	0	2.1	8.0	2.88	1.05	0.167
			1	1.5	7.0	3.22	0.98	
		Left	0	1.8	5.5	2.89	0.69	0.927
			1	1.6	5.3	2.91	0.81	
	CB	Right	0	4.5	13.5	10.08	1.98	0.240
			1	3.8	13.3	9.45	2.46	
		Left	0	4.3	12.2	9.44	1.76	0.990
			1	5.4	15.3	9.45	2.21	

**Table:** (c)

Position		Side	Gender	Minimum	Maximum	Mean	SD	*P* value
P1	B	Right	0	4.3	10.6	6.62	1.24	0.484
			1	3.8	10.3	6.34	1.56	
		Left	0	3.6	8.4	5.82	1.58	0.644
			1	3.5	8.4	6.02	1.42	
	L	Right	0	0.9	4.9	1.99	1.03	0.301
			1	1.5	3.4	2.26	0.67	
		Left	0	1.1	5.8	1.88	0.89	0.054
			1	0.9	7.6	2.52	1.40	
	I	Right	0	5.2	13.0	7.37	1.38	0.186
			1	3.8	12.1	6.72	2.00	
		Left	0	3.4	13.0	7.28	2.10	0.052
			1	1.7	8.5	6.13	1.87	
	S	Right	0	2.3	5.1	3.47	0.71	0.452
			1	2.0	5.2	3.32	0.66	
		Left	0	2.4	5.9	3.46	0.68	0.474
			1	1.7	4.6	3.32	0.61	
	CB	Right	0	11.3	20.9	15.68	2.42	0.847
			1	11.7	19.7	15.81	2.24	
		Left	0	10.1	18.3	14.93	2.35	0.398
			1	8.7	17.4	14.38	2.12	

P2	B	Right	0	2.7	9.1	5.70	1.62	0.534
			1	3.0	12.8	6.03	2.15	
		Left	0	2.2	8.5	5.27	1.49	0.388
			1	3.6	8.6	5.62	1.31	
	L	Right	0	1.0	4.3	1.96	0.85	0.514
			1	0.8	4.0	2.12	0.87	
		Left	0	1.0	4.8	2.12	0.80	0.685
			1	0.8	4.3	2.22	0.99	
	I	Right	0	4.0	14.5	8.09	1.71	0.076
			1	3.9	10.1	7.21	1.67	
		Left	0	4.8	12.1	7.80	1.89	0.184
			1	4.1	9.2	7.12	1.57	
	S	Right	0	2.5	4.1	3.21	0.56	0.623
			1	1.9	4.5	3.29	0.64	
		Left	0	2.9	4.4	3.49	0.56	0.135
			1	2.0	4.2	3.23	0.63	
	CB	Right	0	7.3	15.5	11.38	1.93	0.979
			1	8.6	14.9	11.39	1.91	
		Left	0	6.5	16.2	11.02	2.68	0.610
			1	8.9	13.6	10.70	1.29	

P3	B	Right	0	1.3	8.5	3.86	1.92	0.360
			1	1.5	9.4	4.38	2.05	
		Left	0	1.1	8.7	3.60	1.99	0.350
			1	0.8	9.4	4.13	1.95	
	L	Right	0	0.7	5.2	2.42	1.18	0.984
			1	0.8	6.5	2.43	1.62	
		Left	0	0.8	5.3	2.92	1.24	0.483
			1	0.9	5.9	2.63	1.65	
	I	Right	0	3.0	18.2	11.88	2.55	0.012*
			1	5.7	14.4	9.95	2.59	
		Left	0	6.8	16.0	11.07	2.82	0.525
			1	5.5	15.6	10.58	2.54	
	S	Right	0	2.1	8.0	2.91	1.08	0.772
			1	1.5	4.1	2.83	0.82	
		Left	0	1.8	4.3	2.81	0.57	0.744
			1	1.3	3.9	2.75	0.70	
	CB	Right	0	7.3	11.6	9.48	1.52	0.918
			1	6.1	12.4	9.53	1.94	
		Left	0	5.7	18.5	9.92	2.78	0.427
			1	5.8	14.1	9.32	2.37	

Groups: 1: women, 0: men; *: statistical significance; SD: standard deviation.
